# Inflammation-Based Hematological Indices (NLR, PLR, LMR) in Pancreatic Cancer: Implications for Laboratory Diagnostics and Clinical Interpretation

**DOI:** 10.3390/cancers18142313

**Published:** 2026-07-17

**Authors:** Iwona Zawistowska, Blanka Wolszczak-Biedrzycka, Tomasz Kukliński, Violetta Dymicka-Piekarska, Justyna Dorf

**Affiliations:** 1 Medical Laboratory Diagnostic, Polish Red Cross Memorial Municipal Hospital, Henryka Sienkiewicza 79 St., 15-003 Bialystok, Poland; 2 Department of Psychology and Sociology of Health and Public Health, University of Warmia and Mazury in Olsztyn, Warszawska 30 St., 10-082 Olsztyn, Poland; blanka.wolszczak@uwm.edu.pl; 3 Department of Internal Medicine and Gastroenterology, Independent Public Health Care Center of the Ministry of Internal Affairs and Administration in Białystok Marian Zyndram–Kościałkowski, Fabryczna 27 St., 15-471 Bialystok, Poland; tomasz.kuklinskihtc@gmail.com; 4 Department of Clinical Laboratory Diagnostics, Medical University of Bialystok, Waszyngtona 15a St., 15-269 Bialystok, Poland; violetta.dymicka-piekarska@umb.edu.pl

**Keywords:** LMR, NLR, pancreatic cancer, PDAC, PLR

## Abstract

*Objectives*: Pancreatic cancer remains one of the most aggressive malignancies, characterized by late diagnosis, limited therapeutic options, and poor survival outcomes. Increasing evidence indicates that systemic inflammation and tumor microenvironment interactions play a crucial role in disease progression and patient prognosis. This review aims to summarize current evidence on the clinical utility of inflammation-based hematological indices derived from complete blood count (CBC), including the neutrophil-to-lymphocyte ratio (NLR), lymphocyte-to-monocyte ratio (LMR), and platelet-to-lymphocyte ratio (PLR), in pancreatic cancer. *Content*: Available data consistently demonstrate that elevated NLR and PLR, as well as decreased LMR, are associated with poorer overall survival, more aggressive disease phenotype, and reduced response to therapy. Among these indices, NLR appears to be the most robust and widely validated prognostic marker. Its clinical value is enhanced when combined with markers such as CA 19-9. LMR reflects the balance between host immune response and monocyte-derived tumor-promoting activity, while PLR highlights the role of platelets in tumor progression, angiogenesis, and immune evasion. Despite their potential, the routine clinical implementation of these indices is limited by the lack of standardized cut-off values, variability between patient populations, and susceptibility to confounding factors such as inflammatory conditions. *Summary and outlook*: In this article we show that inflammation-based hematological indices represent inexpensive, accessible, and promising tools for laboratory diagnostics and prognostic assessment in pancreatic cancer. Their integration with clinical, biochemical, and molecular data may improve risk stratification and support personalized therapeutic strategies; however, further large-scale prospective studies are required to establish their standardized clinical use.

## Simple Summary

Pancreatic cancer is one of the most dangerous and common cancers. Its invasiveness and high metastatic potential result from the specific tumor microenvironment, in which inflammation plays a key role. Prompt diagnosis is crucial, allowing for the implementation of appropriate treatment at an early stage. The aim of our review is to assess the clinical utility of hematological indicators related to inflammation found in routine blood tests, such as complete peripheral blood counts: the neutrophil-to-lymphocyte ratio (NLR), the lymphocyte-to-monocyte ratio (LMR), and the platelet-to-lymphocyte ratio (PLR). We have demonstrated that elevated NLRs and PLRs, as well as decreased LMR, characterize patients with poorer prognosis and overall survival. Due to their low cost and widespread availability, these indicators may become valuable prognostic biomarkers for pancreatic cancer, supporting faster therapeutic decision-making and improving patient prognosis.

## 1. Introduction

Pancreatic cancer is one of the most common cancers, ranking fifth in terms of cancer incidence among all cancer types. Every year, an average of 459,000 new cases of pancreatic cancer are observed, albeit the number of deaths is estimated at 227,000. Consequently, the mortality-to-morbidity ratio remains dismally high, reaching 98% [1]. The 5-year overall survival rate for pancreatic cancer patients is reported to be less than 7% [2]. Pancreatic cancer occurs significantly more frequently in countries with a high Human Development Index (HDI). The highest incidence is observed in the countries of Western Europe (8.3/100,000), North America (7.6/100,000), Central and Eastern Europe (7.5/100,000), Northern Europe (7.3/100,000), and Southern Europe (7.2/100,000). The lowest incidence of pancreatic cancer is reported in East and Southeast Asia (<1.5/100,000) [3]. Risk factors encompass smoking, age over 55 years, diabetes, obesity, chronic pancreatitis, liver cirrhosis, Helicobacter pylori infection, occupational exposure to chemicals in dry cleaning and the metal industry, family history, and genetic predisposition, including genetic mutations or association with syndromes such as Lynch syndrome, Peutz–Jeghers syndrome, von Hippel–Lindau syndrome, and MEN1. Furthermore, factors that increase the risk of pancreatic cancer include excessive consumption of coffee, red wine, and high consumption of red meat [4].

Early detection of pancreatic cancer is crucial for patient survival, as the disease is usually diagnosed at an advanced stage, when treatment becomes significantly less effective. Treatment for pancreatic cancer is extremely difficult and often does not bring the expected results. The most effective treatment method remains surgical resection of the tumor. Depending on the location of the lesion, various procedures are used: pancreatoduodenectomy (Whipple procedure) for tumors located in the head of the pancreas, or distal pancreatectomy for lesions in the body or tail of the pancreas [5]. During these procedures, the head of the pancreas and the duodenum are removed, a partial circumferential resection of the pancreas is performed, or—in some cases—a complete removal of the pancreas and the duodenum is performed [6]. Unfortunately, approximately 80% of patients develop metastases within two years [7]. Neoadjuvant treatment is increasingly used in patients with resectable pancreatic adenocarcinoma. The goal of such therapy is to improve the patient’s general condition, enable safe chemotherapy, and complete it within 4–6 months. This method yields the best results in patients with non-metastatic pancreatic cancer [8]. Basic treatment regimens include FOLFIRINOX and a combination of gemcitabine with protein-bound paclitaxel. FOLFIRINOX is a combination therapy of 5-fluorouracil, oxaliplatin, and irinotecan [9]. Due to its high toxicity profile, this regimen is primarily used in younger individuals with an excellent performance status. In contrast, for older patients, gemcitabine combined with protein-bound paclitaxel is preferred [10].

### Tumour Microenvironment

The tumour microenvironment of pancreatic ductal adenocarcinoma (PDAC) is characterized by a highly complex architecture and unique biological dynamics, encompassing both intense stromal proliferation and extensive metabolic adaptation that allows cancer cells to survive in an environment of extreme hypoxia. The dominant presence of a mesenchymal component is one of the key features distinguishing PDAC from other solid tumors. Mesenchymal cells act as regulators of paracrine signalling, modulating the phenotype and behaviour of tumor epithelial cells through complex cytokine and chemokine interactions [11].

A significant element of the PDAC microenvironment is its spatial heterogeneity, determined by the presence of diverse cell populations, such as cancer-associated fibroblasts (CAFs), immune cells, endothelial cells, peripheral neurons, and a dense extracellular matrix (ECM). They may promote metastasis by inhibiting the synthesis of proinflammatory cytokines. CAFs also produce basement membrane components, metalloproteinases (MMP-1, MMP-7, MMP-9), and VEGF, a potent proangiogenic factor [11]. Concurrently, ECM plays a pivotal role in cancer progression due to its structural and signalling properties. ECM-integrin interactions promote the proliferation and migration of cancer cells, while cytokines, chemokines, and growth factors sequestered in the ECM increase their invasiveness and metastatic potential.

Fibrillar collagens constitute the dominant group of ECM proteins in PDAC and are primarily synthesized by activated CAFs in direct contact with cancer cells. Excessive accumulation of collagen and other ECM components leads to increased tissue stiffness, impaired tumor perfusion, and limited drug diffusion, contributing to the development of a chemoresistant phenotype. Furthermore, cancer cells demonstrate the ability to scavenge and metabolize components of the microenvironment, enabling them to adapt to extreme glucose and oxygen deprivation and to evade immune surveillance [12]. These mechanisms are key determinants of PDAC resistance to conventional chemotherapy regimens.

Nerve–tumor interactions represent an important yet often underappreciated component of the tumor microenvironment. These interactions are mediated by a variety of signaling molecules and molecular pathways that influence tumor growth and progression. Intratumoral nerves can regulate angiogenesis through both direct and indirect mechanisms. Direct effects are mediated by neurotransmitters acting on receptors expressed by cancer cells in a tumor type-specific manner. In PDAC, norepinephrine plays a particularly important role by activating β2-adrenergic receptors, thereby enhancing glycolytic metabolism, tumor cell proliferation, and invasive potential. Indirect regulation occurs through the release of neuropeptides and chemokines by nerve fibers, which recruit and modulate immune cells within the tumor microenvironment, ultimately promoting tumor growth and metastatic dissemination. Recent studies have also introduced the concept of an “immunological engram,” suggesting that neural circuits in the brain may influence immune function and contribute to long-term systemic immune regulation. Furthermore, pancreatic cancer cells secrete neurotrophic factors that stimulate intratumoral nerve growth, a process associated with disease progression, tumor heterogeneity, and poor clinical outcomes. Collectively, the dynamic crosstalk between neural elements and other components of the tumor microenvironment supports tumor development, progression, and metastasis. These findings have generated interest in the potential therapeutic use of neuroactive agents, including strategies combining neural modulation or denervation with immunotherapy; however, further investigation is required before such approaches can be implemented in clinical practice [13].

In the context of PDAC treatment, increasing attention is being paid to targeted therapies and immunomodulatory approaches that can potentially overcome microenvironmental barriers to drug resistance. A thorough understanding of the biology of the stroma and its interactions with cancer cells is fundamental to developing more effective therapeutic strategies [14]. Spatial omics technologies represent an emerging approach for investigating the tumor microenvironment and elucidating mechanisms underlying therapeutic resistance in cancer. Unlike conventional molecular profiling techniques, spatial omics integrates molecular information with its precise spatial localization within biological tissues, enabling multidimensional analysis of cellular interactions and tissue architecture. By providing a three-dimensional perspective of the tumor ecosystem, this technology has significantly advanced our understanding of pancreatic cancer biology. Recent studies have demonstrated that resistance to therapy is associated with distinct structural and functional domains within the pancreatic tumor microenvironment. Factors contributing to immune remodeling include reduced glucose availability in specific microenvironmental regions and the accumulation of immunomodulatory metabolites, both of which may impair CD8+ T-cell function and promote immune evasion. Importantly, spatial omics supports the development of precision oncology by transforming conventional molecular data into detailed spatial maps, allowing more accurate prognostic assessment and identification of patient-specific therapeutic targets [15].

Pancreatic ductal adenocarcinoma (PDAC) also harbors a complex intratumoral microbiome that interacts closely with host metabolic and immune pathways. Proteobacteria and Firmicutes are among the predominant bacterial phyla detected within PDAC tissues. These microorganisms may establish symbiotic interactions with resident fungi, contributing to chemoresistance and metabolic reprogramming of tumor cells. Understanding the biological interactions between microbial communities and the tumor microenvironment is increasingly recognized as an important step toward the development of personalized therapeutic strategies. Notably, microbial dysbiosis in PDAC differs according to anatomical location, with distinct microbial profiles identified in the oral cavity, gastrointestinal tract, and tumor tissue. Such findings have raised interest in microbiome-targeted interventions, including selective antimicrobial therapy, probiotics, and microbiota modulation [16].

The oral microbiome has been associated with pancreatic cancer risk and treatment resistance, particularly through the presence of *Porphyromonas gingivalis*, a pathogen capable of promoting chronic inflammation and facilitating oncogenic processes. In addition, the oral microbiota constitutes an important reservoir of antibiotic-resistance genes, which may further complicate treatment strategies [16].

The gut microbiome has also been implicated in systemic chemotherapy resistance. Bacterial genera such as Veillonella, Streptococcus, and Enterococcus can activate inflammatory pathways through the production of lipopolysaccharide (LPS) and other pro-inflammatory mediators. Furthermore, depletion of beneficial butyrate-producing bacteria may impair immune function and contribute to increased resistance to anticancer therapies. Intestinal dysbiosis can compromise epithelial barrier integrity, facilitating the translocation of bacteria and endotoxins into the circulation and thereby promoting systemic inflammation that may adversely affect treatment outcomes [16].

In summary, the microbiome appears to contribute to both intrinsic and acquired chemoresistance in PDAC through multiple mechanisms. These include direct effects, such as drug metabolism and inactivation, as well as indirect effects involving immune microenvironment remodeling, chronic inflammation, and activation of oncogenic signaling pathways. Although microbiota-based therapeutic strategies—including fecal microbiota transplantation, probiotics, and engineered bacterial therapies—have shown promise in preclinical studies, their clinical efficacy and long-term benefits in PDAC remain to be established through further research [16].

**Figure 1:**
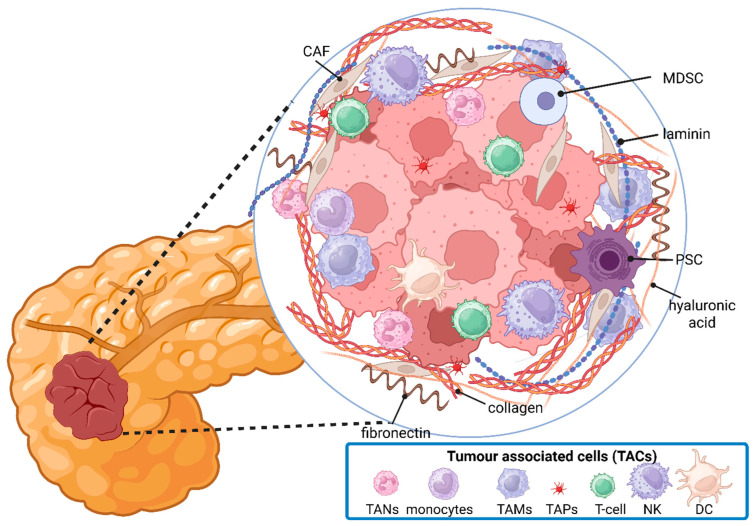
Cellular composition of the tumour microenvironment in PDAC. Abbreviations: TANs—tumour-associated neutrophils, TAM—tumour-associated macrophages, TAPs—tumour-associated platelets, NK—natural killer cells, DC—dendritic cells, CAF—cancer-associated fibroblasts, MDSC—myeloid-derived suppressor cells, PSC—Pancreatic Stellate Cells.

The microenvironment also includes inflammatory cells such as TAMs (Tumor-associated Macrophages), TANs (Tumor-associated Neutrophils), TAPs (Tumor-associated Platelets), myeloid-derived suppressor cells (MDSCs), dendritic cells (DCs), natural killer cells (NK), T and B lymphocytes (Figure 1).

B and T lymphocytes can form clusters (Tertiary Lymphoid Structures, TLS) in tumors, including PDAC. This is associated with the presence of a larger number of lymphocytes in the stroma and, therefore, a better prognosis for the patient. Meanwhile, lymphocytopenia is characteristic of pancreatic cancer, which significantly weakens the immune response [17]. Lymphocytes are essential for a proper immune response against cancer cells. Regulatory T cells (Tregs), a specialized subset of CD4+ T lymphocytes, also play an important role in the pancreatic cancer microenvironment. These cells suppress antitumor immune responses by inhibiting the activity of effector T lymphocytes, natural killer cells, and antigen-presenting cells. Increased infiltration of Tregs has been reported in PDAC and is associated with an immunosuppressive tumor microenvironment, disease progression, and poorer clinical outcomes [18]. Consequently, an imbalance between cytotoxic T cells and Tregs may contribute to impaired antitumor immunity and unfavorable prognosis in patients with pancreatic cancer. CD4+ T lymphocytes are essential for the activation and maintenance of CD8+ T-cell-mediated antitumor responses. These, in turn, cause apoptosis of cancer cells and exhibit direct cytotoxic effects against them. Notably, in pancreatic cancer, the number of CD4+ T lymphocytes is significantly reduced compared to the number of CD8+ T lymphocytes. An insufficient presence of lymphocytes leads to a weakened immune response against cancer cells, which is consequently associated with a poorer prognosis [17].

Monocytes represent another crucial component of the immune system. They are produced in the bone marrow and then migrate through the bloodstream to tissues, where they transform into macrophages and dendritic cells. The role of a macrophage is to engulf foreign cells, kill them, and enhance the immune response [19]. In pancreatic cancer, tumor-associated macrophages (TAMs), derived primarily from peripheral blood monocytes, support the proliferation of cancer cells and exhibit strong immunosuppressive effects. They inhibit the activity of antitumor lymphocytes by secreting cytokines, chemokines, and growth factors, as well as modulating the tumor microenvironment to favor its progression [20].

A key component of the PDAC microenvironment that protects cancer cells from the immune system is myeloid-derived suppressor cells (MDSCs). Under physiological conditions, myeloid cells act as a protective barrier against infections, demonstrating strong phagocytic activity, generating an oxygen burst, and releasing proinflammatory cytokines. In pancreatic cancer, however, they undergo pathological remodelling [21]. Instead of supporting the immune response, they begin to act to the benefit of cancer cells, promoting immunosuppression and tumor progression. MDSCs are not present in healthy individuals; they only appear in cancer patients and in states of chronic stress and inflammation. Under such conditions, the immune system is overstimulated, leading to persistent, albeit moderate, myelopoiesis. The most important characteristic of these cells in relation to cancer is their ability to suppress immune responses through processes related to, among others, nitric oxide and cytokine production. MDSCs also contribute to cancer progression by remodeling the tumor microenvironment and supporting angiogenesis. This occurs, inter alia, through the secretion of VEGF and metalloproteinase-9, which promote the formation of new blood vessels and facilitate tumor invasion [19].

Interestingly, the tumor mass itself is composed of, among other things, neutrophils (tumor-associated neutrophils, TANs). They play a pivotal role in acute and chronic inflammation through phagocytosis, intracellular degradation, and, most importantly, the release of enzymes and active substances from their granules [22]. An increased number of neutrophils may promote the progression of pancreatic cancer by providing appropriate conditions for tumor growth. Neutrophils secrete, among others, tumor necrosis factor (TNF-α) and vascular endothelial growth factor (VEGF), which is a proangiogenic factor [17]. TANs induce tumor growth and metastasis through the production of chemokines and inflammatory factors such as matrix metalloproteinase 9 (MMP-9) and interleukin IL-17. Furthermore, TANs produce ROS, i.e., reactive oxygen species, and intermediate reactive nitrogen species. They cause significant DNA damage, which leads to cancer progression [22,23].

Platelets also form the tumor microenvironment of pancreatic cancer. They participate in the first phase of coagulation by forming a platelet plug and also produce platelet factor 3. An excessively low platelet count is called thrombocytopenia and occurs when there is decreased production by the bone marrow, increased utilization in peripheral blood, or increased destruction by the spleen. An elevated platelet count can be primarily caused by myeloproliferative diseases or splenic resection [24]. In pancreatic cancer, platelets actively contribute to tumor progression by releasing numerous growth factors, including vascular endothelial growth factor (VEGF) and platelet-derived growth factor (PDGF), which promote angiogenesis, tumor growth, and metastatic dissemination [25]. In addition, pancreatic cancer cells can induce a prothrombotic state through increased expression of tissue factor (TF), which binds factor VIIa and initiates the coagulation cascade. This process promotes tumor cell-induced platelet aggregation (TCIPA), facilitating interactions between circulating tumor cells and platelets. The resulting platelet–tumor cell complexes protect malignant cells from immune surveillance, enhance their survival in the bloodstream, and promote metastatic spread to distant organs [26]. Activated platelets also release TGF-β (transforming growth factor-β). Whereas this activates the NK-κB signalling pathway in transformed cells and stimulates tumor progression, among other things, by initiating metastasis [27]. The binding of platelets to tumor cells—the formation of TAPs (Tumor-Associated Platelets)—is facilitated by receptors and molecules present on the platelet surface, such as GP Ib-IX-V, GP IIb-IIIa, GP V, P-selectin, and CLEC-2 (C-type lectin receptor 2) [11].

Cells belonging to the microenvironment play an important role in inflammation, which in turn promotes the development of PDAC. It results from the body’s response to an invading pathogen or other damaging stimuli, leading to the activation of defense mechanisms. Lymphocytes are responsible for the formation of immunological memory and the onset of healing. It is also possible that these cells are unable to contain the pathogen, and chronic inflammation develops. The most characteristic feature of this phase is the massive infiltration of leukocytes into damaged tissues, which in turn may promote neoplastic transformation [23]. Lymphocytes constitute one of the largest subgroups of infiltrating leukocytes. This is crucial because the phenotype of these cells determines treatment outcomes. Tumor-infiltrating lymphocytes secrete various cytokines, including interleukin-4 (IL-4), which has been reported to exert pro-invasive effects on pancreatic cancer cells and may contribute to tumor progression [28]. Inflammatory mediators such as IL-6 and IL-23 promote tumor development by activating transcription factors—nuclear factor kappa light chain enhancer of activated B lymphocytes (NF-κB) and signal transducer and activator of transcription 3 (STAT3). They are responsible for regulating genes responsible for, among other things, immune evasion, thus leading to tumor progression. NF-κB is also responsible for communication between immune cells and the tumor. Furthermore, NF-κB can inhibit inflammatory macrophages (Mic-1) by directly regulating growth and differentiation factor 15 (GDF-15) [29]. Myeloid cells can also infiltrate damaged tissues, just like lymphocytes. Macrophages and mast cells, in turn, release proinflammatory cytokines such as TNF-α, IL-1β, and IL-6, as well as chemokines such as IL-8, MCP-1, and MIP-1α, in response to threats. These mediators initiate and maintain inflammation, which, if chronic, can promote malignant transformation and the progression of pancreatic cancer. Their further role is also to recruit neutrophils to the site of infection, as they are the first line of defense [20]. They are characterized by rapid migration to the site of infection and the ability to eliminate the pathogen independently. Meanwhile, TNF-α and IL-6 enhance epithelial cell proliferation, which facilitates the multiplication of cancer cells. This mechanism is further supported by stromal cells and immune cells recruited to the inflamed site. Another cytokine present in the tumor microenvironment and exerting a supportive effect on tumor development is Il-1-α [30]. Cancer cells expressing this cytokine create an inflammatory profile in PSCs (Pancreatic Stellate Cells). Subsequently, these cells induce PDAC cell migration upon activation by Il-1-α. Furthermore, PSCs then exhibit increased expression of MMP-1 (matrix metalloproteinase-1) and MMP-3 (matrix metalloproteinase-3) and decreased expression of TIMP3 (tissue inhibitor of metalloproteinases 3). This increases the proteolytic activity of TIMP3 and leads to tumor stromal remodeling. Interestingly, the TLR (toll-like receptor) receptor, or rather its ligation, TLR9, also stimulates epithelial cell proliferation in PSCs. TLRs support the inflammatory microenvironment and are induced by DAMPs (damage-associated molecular patterns) produced in response to pancreatitis [31].

The enzyme linking chronic pancreatic inflammation with cancer is cyclooxygenase-2 (COX-2) [32]. The COX-2 promoter has been shown to contain numerous binding sites for NF-κB and other transcription factors [31].

Furthermore, inflammation induces carcinogenesis through the production of reactive oxygen species (ROS) and reactive nitrogen species (RNS). These are produced by activated inflammatory cells—neutrophils and macrophages. Excessively high concentrations of ROS and RNS cause oxidative damage to DNA, lipids, and proteins, resulting in neoplastic transformation [33]. In summary, inflammation plays a significant role in tumor progression, including its initiation, promotion, malignant conversion, invasion, and metastasis.

## 2. Methods

### 2.1. Literature Search and Selection Criteria

This systematic literature review was conducted between 9 January 2026, and 18 April 2026, and aimed to evaluate the clinical utility of inflammation-related hematological indicators (NLR, LMR, and PLR) as prognostic biomarkers in pancreatic cancer.

A comprehensive literature search was performed using PubMed, Wiley Online Library, and SpringerLink databases. The search strategy included the following terms: (“NLR” OR “neutrophil-to-lymphocyte ratio” OR “PLR” OR “platelet-to-lymphocyte ratio” OR “LMR” OR “lymphocyte-to-monocyte ratio”) AND (“pancreatic cancer” OR “PDAC” OR “pancreatic ductal adenocarcinoma”). Additional keywords related to inflammation, inflammatory markers, diagnostic utility, ROC curve, AUC, and complete blood count were used where appropriate. The final search was conducted on 18 April 2026. Eligible studies included original retrospective and prospective investigations evaluating the clinical significance of NLR, LMR, or PLR in pancreatic cancer. Exclusion criteria comprised studies with insufficient data, unavailable full texts, case reports, editorials, letters to the editor, narrative reviews, studies investigating biomarkers other than NLR, LMR, or PLR, articles with unclear methodology, and publications written in languages other than English. We acknowledge that restricting the search to English-language publications may have introduced language bias and may have resulted in the omission of potentially relevant studies.

Data extraction was performed independently by three reviewers and included information on study design, sample size, patient characteristics, evaluated inflammatory biomarker (NLR, LMR, or PLR), cut-off value, reported outcomes, and principal findings. Any discrepancies were resolved through discussion and consensus.

Methodological quality and potential sources of bias were assessed qualitatively by evaluating study design, patient selection methods, sample size, outcome reporting, adequacy of reported clinical data, and consistency of findings with the existing literature. Particular attention was paid to potential sources of bias, including retrospective study design, heterogeneous patient populations, differences in treatment strategies, and variability in cut-off values used for inflammatory indices. Due to the heterogeneity of the included studies and the narrative nature of the evidence synthesis, no formal risk-of-bias assessment tool (e.g., Newcastle–Ottawa Scale, ROBINS-I, JBI Critical Appraisal Tools, or Cochrane Risk of Bias tool) was applied. The limitations associated with this approach were considered during data interpretation and formulation of conclusions.

**Figure 2:**
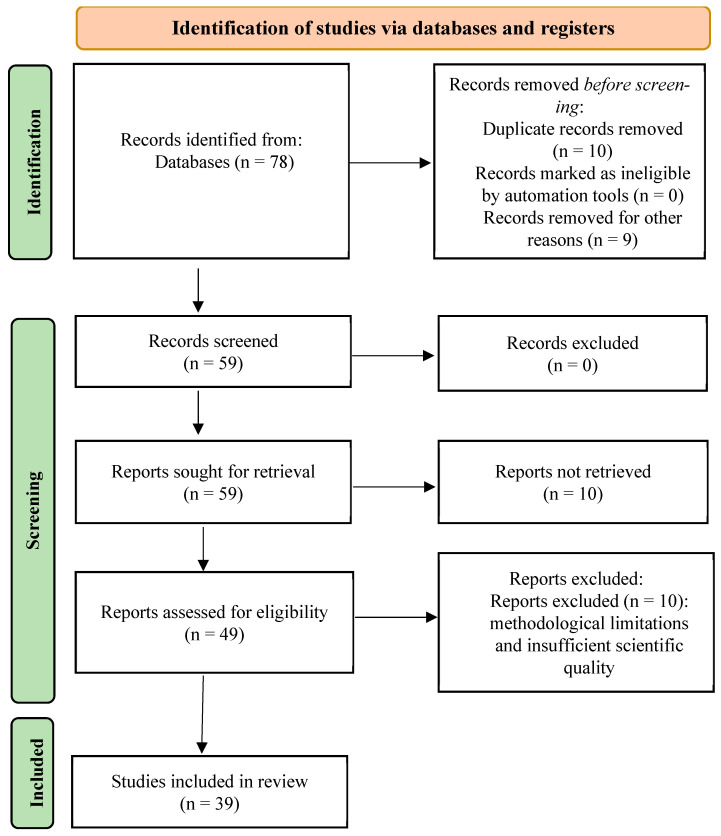
PRISMA flow diagram for studies evaluating inflammatory markers (NLR, PLR, LMR) in pancreatic cancer.

The initial search identified 78 records. After removal of 10 duplicates, 68 studies remained for screening. Nine articles were excluded based on irrelevant biomarkers (other than NLR, LMR, or PLR), and 10 studies were excluded due to lack of available full texts. A total of 49 full-text articles were assessed for eligibility. Of these, 10 studies were excluded due to insufficient methodological quality (n = 2), insufficient scientific value (n = 3), or unclear study design (n = 5). Ultimately, 39 studies were included in the final analysis. The study identification and selection process is summarized in Figure 2.

This review was conducted following PRISMA recommendations for literature identification, screening, study selection, and reporting.

The review protocol was not registered in any publicly available database.

### 2.2. Complete Blood Count

In the context of the described inflammatory mechanisms associated with neoplastic transformation, it is crucial to use simple, non-invasive tools to assess the intensity and nature of this condition. One of the most easily accessible and at the same time very informative tests is peripheral blood count, which, as a cost-effective, widely available and routinely performed test, plays an important role in the diagnosis and monitoring of the course of cancer. Changes in morphological parameters often reflect the body’s systemic inflammatory response to a developing tumor, hematopoietic disorders, or the impact of anticancer treatment. A growing body of data indicates that traditionally determined parameters and simple inflammatory markers derived from them (such as NLR, LMR, or PLR) may have prognostic and predictive value in various types of cancer, including pancreatic cancer. However, despite promising research results, currently used markers are not always characterized by sufficient clinical sensitivity and specificity. Their baseline values can be confounding by concomitant factors, such as infections, inflammatory diseases, or autoimmune disorders. Therefore, there is a clear need to develop new, more precise markers based on blood counts or their extended analysis, which would better reflect the interactions between the immune system and the tumor microenvironment. The development of such parameters could contribute to earlier disease detection, more accurate prognosis assessment, and better personalization of cancer treatment. Considering the growing importance of peripheral blood counts as a simple and readily available tool supporting the diagnosis and assessment of prognosis in cancer patients, as well as the limitations of currently used parameters, this paper reviews the literature on the clinical significance of selected inflammatory markers. This review focused on the assessment of the neutrophil-to-lymphocyte ratio (NLR), lymphocyte-to-monocyte ratio (LMR), and platelet-to-lymphocyte ratio (PLR) in patients with pancreatic cancer. The aim of the analysis was to collect and organize available data regarding their diagnostic and prognostic value and to assess the potential of these markers as supportive elements in assessing the course of the disease.

## 3. NLR—Neutrophil to Lymphocyte Ratio

The diagnostic value of this marker stems from the key role that infiltrating neutrophils and lymphocytes play in the pancreatic tumor microenvironment. In the course of cancer, a systemic inflammatory response develops, promoting increased neutrophil infiltration into the tumor microenvironment. These neutrophils secrete numerous proinflammatory and proangiogenic factors, such as IL-2, IL-6, IL-10, TNF-α, and VEGF, which create an environment conducive to cancer progression. Meanwhile, elevated levels of TNF-α and IL-10 contribute to decreased lymphocyte counts and impaired function, further weakening the antitumor response. Consequently, high neutrophil counts combined with reduced lymphocyte counts result in an elevated NLR, which may be a valuable prognostic marker in patients with pancreatic cancer. Furthermore, modulating the systemic inflammatory response may be a promising therapeutic strategy, especially in patients with pancreatic cancer who have an elevated NLR.

Zi-jun Xiang et al. demonstrated that NLR is a significant and independent prognostic factor in patients with pancreatic cancer after surgical treatment. An elevated NLR (>2.5) was associated with significantly compromised overall survival and progression-free survival, while a low NLR (<2.5) correlated with a better prognosis. Importantly, NLR proved to be a better predictor of survival than the CA19-9 marker, and its value also reflected the status of the patients’ immune response. The diagnostic value of NLR was very good—0.840 [34] (Table 1). Zhou et al. also demonstrated that a high NLR (>2.9) is a strong and independent predictor of poor prognosis in patients with pancreatic cancer after both surgical treatment and neoadjuvant chemotherapy. The diagnostic value was 0.761 with a specificity of 95.6% and a sensitivity of 48.9%, which indicates good diagnostic usefulness of this parameter. Patients with an elevated NLR had significantly compromised overall survival. In addition, Zhou et al. demonstrated that NLR is an even better marker of poorer survival in patients when combined with CA 19-9 assessment. The authors emphasize that the easy availability and low cost of NLR testing make it a valuable tool for assessing prognosis and identifying patients at high risk of an unfavorable disease course, although further prospective studies are necessary to standardize it [35]. Similarly, studies by Asaoka et al. demonstrated that an elevated NLR (>2.7) is a significant diagnostic marker in patients with unresectable pancreatic cancer. NLR significantly correlates with shorter patient survival and may serve as a useful predictor before surgery in patients with pancreatic cancer. The authors also emphasize the low cost of the test and the easy availability of this parameter [36]. In an analysis conducted by Chen Y et al. patients with NLR >2.78 had substantially worse overall survival. The authors demonstrated that an increase in this parameter significantly correlates with increased mortality among patients. This may indicate inappropriate treatment or further progression of the cancer despite chemotherapy. This explains why the NLR marker is so important in monitoring patients undergoing chemotherapy [37]. Cheng H et al. observed that an NLR >2 significantly correlates with poorer survival in patients with pancreatic cancer. Furthermore, they demonstrated that pancreatic cancer patients with a high NLR and an increased percentage of Treg cells are more than 3.5 times more likely to die earlier than patients with a low NLR and a low percentage of Treg cells. The researchers also demonstrated that elevated NLR and Treg cell values occur in patients with tumors located in the distal part of the pancreas, suggesting that these tumors have a poorer prognosis [38]. Further studies demonstrating the importance of NLR as a prognostic predictor were conducted by Umut Varol et al. They demonstrated that the median survival in patients with a high NLR (>2.7) was half that of patients with NLR < 2.7 [39]. Xue P et al. demonstrated that the aforementioned parameter can serve as an indicator of the risk of death in patients with pancreatic cancer who are in poor condition. The authors note that NLR (>5) may play a significant role in emergency medicine and intensive care to predict the prognosis of these patients [40]. Naoto Iwai et al. demonstrated that a high NLR (>3.74) is a significant predictor of mortality in patients with inoperable pancreatic cancer (AUC = 0.792). It plays a particularly important role in immune-mediated or inflammatory diseases [41]. Ventriglia J et al. indicated that a high NLR at the beginning of treatment (≥5) is associated with a shorter time to disease progression, shortened overall survival, and remains an independent predictor of poor prognosis in patients with metastatic pancreatic cancer treated with gemcitabine and nab-paclitaxel [42]. Researchers Maloney S et al. showed that NLR > 5 is associated with significantly shorter survival time in patients after resection of pancreatic cancer. In such patients, therapy may be ineffective, and disease progression may be more rapid. In their study, the authors reported that neoadjuvant treatment yields better results in patients with a lower NLR (<5) [43]. Hacker D et al. demonstrated that a preoperative NLR > 3.2 was associated with significantly compromised overall survival in patients with pancreatic cancer compared to those with an NLR ≤3.2 (median OS: 17.1 vs. 27.9 months). An NLR > 3.2 remained an independent prognostic factor in multivariate analysis (HR ≈ 1.6), and its significance was particularly pronounced in patients with locally advanced tumors (pT3/4) and lymph node involvement. These results confirm that an elevated NLR reflects a more aggressive disease course and may be a useful preoperative marker for prognostic stratification in PDAC [44]. Wnuk J et al. found that NLR > 6.63 is a predictor of shorter survival time in patients with pancreatic cancer. NLR retained its prognostic significance in both univariate and multivariate analyses, alongside classic markers such as CRP and CA 19-9. These results confirm that elevated NLR reflects increased systemic inflammation and is associated with a more aggressive disease course [45]. Szkandera J et al. demonstrated that elevated neutrophil-lymphocyte ratio, including the derived NLR (dNLR ≥ 2.3), is significantly associated with poorer survival in patients with pancreatic cancer. High dNLR values correlated with more advanced disease stages, higher CA 19-9 levels, and a lower percentage of patients qualified for surgical treatment. In multivariate analysis, dNLR retained its significance as an independent prognostic factor, confirming the value of peripheral blood inflammatory markers as simple and accessible prognostic markers in pancreatic cancer. These authors suggest the possibility of validating the parameter and using it in routine clinical diagnostics [5]. Sakamoto T et al. demonstrated that an NLR value >1.69 indicates significantly worse 2-year survival (3.4% vs. 40.2%, *p* < 0.001). In multivariate analysis, the combination of high NLR and high CA 19-9 levels proved to be an independent predictor of poor prognosis, and the ROC plot for the combination of NLR + CA19-9 showed an AUC of 0.772 (*p* < 0.001), outperforming the value of each variable individually. The authors also emphasized the easy availability of the test, which may make it a routinely assessed biomarker of prognosis in patients with pancreatic cancer [46]. In a retrospective study by Yang M et al., a preoperative NLR ≥3.29 was associated with significantly worse overall and disease-free survival in patients with pancreatic cancer (*p* = 0.003 and *p* = 0.044, respectively). High NLR also correlated with an increased frequency of the adenosquamous carcinoma (ASC) subtype (*p* = 0.009) and a stronger neutrophil infiltration (CD15^+^, *p* = 0.0198) and a reduced number of T lymphocytes (CD8+, borderline difference, *p* = 0.0463) in the tumor tissue. The results suggest that an elevated NLR reflects a suppressive immune microenvironment and the malignant phenotype of PDAC, making it a simple and mechanistically sound prognostic marker [47].

Hasegawa S et al. demonstrated that NLR can also be helpful in selecting treatment. Analyses showed that in the group with a higher NLR (>2.2), the effect of preoperative chemotherapy was highly unsatisfactory (good outcome rate: 44.1% vs. 9.1%, *p* = 0.00699). In multivariate analysis, an NLR ≥2.2 remained an independent predictor of an ineffective pathological response (OR = 5.35, *p* = 0.0257). According to the results, patients had a five-fold lower chance of treatment success. Additionally, higher NLR values were associated with visible neutrophilic tumor infiltration and the absence of lymphoid follicles, suggesting that this index reflects suppression of the immune response and may serve as a simple marker identifying patients less responsive to preoperative treatment. Therefore, using this index to assess treatment efficacy seems crucial [48]. In the study by Kitsugi K et al., [49] the cutoff value of NLR determined using the ROC method was 3.10, which allowed for the identification of groups of patients at lower and higher risk of an unfavorable disease course, including a poorer response to chemotherapy and compromised overall survival. Patients with NLR <3.10 had a significantly higher response to chemotherapy (OR ≈ 4.9) and longer overall survival (HR ≈ 0.42), regardless of the type of treatment and CA19-9 levels. Patients with NLR <3.10 also experienced a higher rate of conversion to third-line treatment and a longer duration of therapy, which translated into higher survival rates of up to 3 years. All of the above observations are presented in Table 1.

Notwithstanding the above, it should be emphasized that despite the clear prognostic value of NLR, the lack of a uniform cut-off point, ranging from 1.69 to 6.63 in the analyzed studies, currently limits its full standardization and routine clinical use. The considerable variability in reported NLR cut-off values may result from differences in patient populations, disease stage, treatment modalities, study design, sample size, and statistical approaches used to determine optimal thresholds. Consequently, direct comparison between studies remains challenging, and the lack of standardized cut-off values currently limits the routine clinical implementation of this biomarker. Large prospective multicenter studies and future meta-analyses using harmonized methodologies are needed to establish clinically applicable and universally accepted threshold values. Such efforts may also help define the role of NLR in clinical decision-making algorithms for pancreatic cancer.

**Table 1:** NLR in Various Studies.

Authors	Study Group N	Control Group N	NLR Values		*p* Value	Cut-Off	AUC	Specificity (%)	Sensitivity (%)	Observations
			Study	Control						
Zi-jun Xiang et al. 2020 [34]	N = 42	N = 25	>2.5	<2.5	<0.05	2.5	0.840	-	-	- High NLR (>2.5) was associated with poorer survival outcomes in patients with pancreatic cancer. - NLR proved to be a better predictor of survival than CA19-9.
Asaoka T et al. 2016 [36]	Received adjuvant chemotherapy using Gemcitabine or S-1 N = 20	Non-survival N = 26	>2.7	<2.7	0.0048	2.7	0.345	-	-	- High NLR (>2.7) was significantly associated with poorer survival in patients with unresectable pancreatic cancer.
Chen Y et al. 2017 [37]	N = 78	N = 54	>2.78	<2.78	<0.001	2.78	0.634	-	-	- Baseline NLR >2.78 and positive ΔNLR were associated with poorer prognosis and may support treatment monitoring.
Oh D et al. 2018 [50]	N = 14	N = 15	>4	<4	<0.001	4	0.379			- High NLR was significantly associated with poorer overall survival in pancreatic cancer (pooled HR ≈ 1.74), with the strongest prognostic value observed in surgically treated and early-stage patients.
Cheng H et al. 2016 [38]	N = 128	N = 67	>2	<2	0.001	2.6	0.538	-	-	- High NLR (>2.0) was significantly associated with poorer overall survival in patients with resectable pancreatic cancer, particularly when combined with an increased proportion of circulating regulatory T cells (Tregs). - The combined NLR–Treg model provided superior prognostic stratification compared with either parameter alone, identifying patients at markedly higher risk of early mortality.
Umut Varol et al. 2020 [39]	High N = 142	Low N = 49	>2.4	<2.4	<0.0001	4	-	-	-	- Baseline NLR >2.4 was associated with significantly shorter OS (4 vs. 10 months, *p* < 0.0001) and shorter progression-free survival in patients with advanced pancreatic cancer.
Zhou et al. 2021 [35]	High N = 118	Low N = 123	>2.9	<2.9	<0.0001	2.9	0.761	95.6	48.9	- Elevated NLR (>2.90) was associated with poorer DFS and OS in advanced PDAC patients with portal system invasion undergoing curative resection.
P. Xue et al. 2014 [40]	N = 40	N = 212	>5	<5	<0.01	5	-	-	-	- High pretreatment NLR (>5) independently predicted shorter TTF and OS; reduction in NLR during chemotherapy was associated with improved survival.
Varzaru et al. 2024 [51]	N = 74	N = 8	>3.31	<3.31	0.009	3.31	0.331	-	-	- Elevated NLR (≥3.31) was associated with significantly shorter overall survival in patients with pancreatic ductal adenocarcinoma, confirming its value as a prognostic marker of systemic inflammation. - NLR showed no significant correlation with circulating cfDNA, but their combination improved prognostic accuracy, indicating complementary biological information.
Iwai N et al. 2020 [41]	N = 69	N = 49	>3.74	<3.74	<0.001	3.74	0.792	-	-	- Elevated NLR (>3.74) was associated with poorer overall survival in patients with pancreatic cancer, indicating that a heightened systemic inflammatory response predicts worse prognosis. - NLR was identified as an independent prognostic biomarker, suggesting its potential usefulness for risk stratification and outcome prediction in pancreatic cancer.
Ventrigilia J et al. 2018 [42]	High N = 49	Low N = 21	>5	<5	0.005	5	0.631	78%	47%	- In metastatic pancreatic cancer treated with nab-paclitaxel plus gemcitabine, a baseline NLR ≥ 5 independently predicted poorer overall survival, supporting the clinical utility of this cut-off for prognostic stratification.
Maloney Sara et al. 2023 [43]	Surgery patients n = 86	Received neoadjuvant chemotherapy n = 110	>5	<5	0.001	5	-	-	-	- A baseline NLR >5 was associated with markedly reduced overall survival in patients undergoing resection for pancreatic cancer, supporting the use of this predefined cut-off for prognostic stratification.
Hackner, D et al. 2024 [44]	N = 97	N = 110	>3.2	<3.2	<0.001	3.2	0.610	64.8	56.3	- An elevated preoperative NLR (>3.2) was an independent predictor of poorer overall survival after primary resection of pancreatic ductal adenocarcinoma, highlighting its value as a simple prognostic biomarker.
Wnuk J et al. 2025 [45]	CS (clinical stage) IV N = 26	CS III N= 24	3.69	2.46	0.043	6.63	-	-	-	- Patients with a preoperative NLR >3.69 demonstrated significantly reduced overall survival after primary resection of pancreatic ductal adenocarcinoma, indicating that this cut-off provides clinically relevant prognostic stratification.
Szkandera J et al. 2013 [5]	N = 271	N = 203	>2.3	<2.3	0.041	2.3	-	-	-	- A dNLR ≥2.3 was independently associated with poorer cancer-specific survival in pancreatic cancer patients, confirming the prognostic value of a ROC-defined cut-off for risk stratification.
Pointer et al. 2020 [52]	N = 38	N = 239	>5	<5	0.002	4.8	-	-	-	- A preoperative NLR ≥5 was independently associated with worse overall and recurrence-free survival after resection of early-stage pancreatic ductal adenocarcinoma.
Sakamoto et al. 2018 [46]	High N = 36	Low N = 30	>1.69	<1.69	<0.001	1.69	0.647	-	-	- In patients with recurrent pancreatic cancer, an NLR ≥1.69 at the time of recurrence was associated with markedly reduced post-recurrence survival, and its combination with elevated CA19-9 further improved prognostic discrimination.
Yang M. et al.2025 [47]	N = 15	N = 49	>3.29	<3.29	0.003	3.29	0.645	-	-	- A preoperative NLR ≥3.29 identified pancreatic cancers with significantly worse overall and disease-free survival and was strongly associated with a suppressive immune microenvironment and the basal-like molecular subtype.
Hagesawa S et al. 2016 [48]	Evans grade I/IIa n = 40	Evans grade IIb/III n = 16	2.9 ± 1.8	1.9 ± 0.6	0.0481	2.2	0.675	-	-	- NLR ≥ 2.2 independently predicted a poor pathological response to neoadjuvant chemoradiotherapy in pancreatic cancer, indicating that NLR may serve as a predictive biomarker of treatment resistance rather than prognosis alone.
K. Kitsugi et al. 2024 [49]	Response group = 36	Non-response group = 24	2.70	3.49	0.048	3.10	0.652	-	-	- In advanced PDAC, a baseline NLR <3.10 predicted better response to first-line chemotherapy and longer overall survival, supporting its use for treatment stratification.

Taken together, the available evidence suggests that elevated NLR reflects an imbalance between a protumor inflammatory response driven by neutrophils and a weakened antitumor immune response mediated by lymphocytes. This biological imbalance may contribute to tumor progression, treatment resistance, and poorer survival outcomes, providing a mechanistic explanation for the prognostic significance of NLR in pancreatic cancer.

## 4. LMR—Lymphocyte to Monocyte Ratio

This indicator is used to assess the prognosis of patients with pancreatic cancer due to the immunosuppressive effects of monocytes. Monocytes inhibit lymphocyte function by secreting cytokines such as CSF-1, chemokines, and growth factors, leading to lymphocytopenia [53]. CD4+ and CD8+ T lymphocytes are crucial in the antitumor immune response. Their role is to initiate cytotoxic cell death and limit the proliferation and migration of tumor cells. Tumor-associated macrophages (TAMs) derived from monocytes have been found to promote tumor invasion and metastasis through the production of epidermal growth factor (EGF), vascular endothelial growth factor (VEGF), interleukin-6 (IL-6), interleukin-10 (IL-10), and matrix metalloproteinases (MMPs). Thus, a high monocyte count together with a reduced lymphocyte count leads to a reduced LMR value. Understanding the role of LMR in pancreatic cancer may not only help predict prognosis further to supporting the development of new therapeutic strategies that could increase treatment efficacy and improve the quality of life of patients with this cancer.

Stotz et al. demonstrated that higher LMR (>2.8) occurred in patients with less advanced disease and better tumor differentiation, and the prognosis was significantly better than for patients with LMR <2.8. In univariate analysis, higher LMR was associated with better cancer-specific survival with a hazard ratio (HR) of 0.70, suggesting that patients with higher LMR have a better prognosis. The authors suggest that LMR can be used as a simple and inexpensive biomarker in clinical practice to assess the prognosis of patients with pancreatic cancer [54] (Table 2). Maloney S et al. showed that LMR may be a prognostic factor, but only for relapse-free survival (RFS). Interestingly, a ratio value >2.4 indicated a two-fold higher probability of pancreatic cancer recurrence. Unfortunately, LMR did not predict the effects of chemotherapy or overall survival in patients [43]. Li W. et al. demonstrated a significant association between LMR and overall survival. Higher LMR values (>3) were associated with a significantly better prognosis, while a reduced LMR was characteristic of patients with more aggressive and advanced cancer [53]. Lin et al. demonstrated that a higher pretreatment lymphocyte-to-monocyte ratio (>3) is significantly associated with improved survival outcomes in patients with pancreatic cancer. Elevated LMR was linked not only to longer overall survival but also to better disease-free or recurrence-free survival, with consistent effects observed across different treatment modalities, ethnic populations, and LMR cut-off values. These findings support LMR as a robust inflammation-based prognostic biomarker reflecting the host immune status in pancreatic cancer [55]. Li et al. and Lin et al. demonstrated that the lymphocyte-to-monocyte ratio can serve as a significant predictor of overall survival—the higher the ratio, the better the patient’s prognosis [53,55]. Shimizu et al. demonstrated that a reduced LMR index (<3.4) is a negative prognostic marker in patients with inoperable pancreatic cancer undergoing chemotherapy. Analyses demonstrated a relationship between LMR values and patient survival. Patients whose LMR values decreased from >3.4 to <3.4 had significantly lower survival chances. Furthermore, these patients demonstrated a poorer response to chemotherapy, suggesting the importance of monitoring LMR during the course of therapy [56]. Studies by Li H. et al. demonstrated that LMR is a significant and independent prognostic marker in patients with pancreatic cancer. Higher LMR values, most often defined by a cut-off point of ≥3, were associated with significantly better overall survival, whereas lower LMR was associated with a worse prognosis. The prognostic significance of LMR persisted regardless of geographic region, treatment type, and cutoff point used, confirming its stability and potential clinical applicability [57]. Hu R. et al. demonstrated that a reduced lymphocyte-to-monocyte ratio (LMR < 3) is a poor prognostic factor in patients with pancreatic cancer. Low LMR values were significantly associated with shorter overall survival and were more frequently observed in male patients, those with elevated CA19-9 levels, and more advanced disease stages. Among the inflammatory markers analyzed, LMR demonstrated greater prognostic stability, underscoring its potential clinical utility [58]. Xue et al. conducted a validation study assessing the significance of LMR as a prognostic factor in patients undergoing pancreatic cancer resection. Analyses showed that a reduced preoperative LMR (<2.8) was significantly associated with shorter overall survival, and its prognostic significance persisted after adjusting for classic clinicopathological factors in multivariate analysis. Importantly, these results were confirmed in an independent cohort, indicating good reproducibility and prognostic stability of LMR. The authors emphasize that LMR can be a simple and reliable marker for risk stratification in patients after resection of pancreatic cancer [59]. In the study by Pointer et al., no significant association of LMR with overall or recurrence-free survival was found in multivariate analysis, suggesting limited utility of this prognostic parameter in this specific patient population [52]. These observations indicate the need for further research on the role of LMR in pancreatic cancer. All of the above observations are presented in Table 2.

From a mechanistic perspective, a low LMR reflects both reduced lymphocyte-mediated antitumor immunity and increased monocyte-derived tumor-promoting activity. This shift toward an immunosuppressive microenvironment may facilitate tumor progression and explain the association between low LMR and unfavorable clinical outcomes.

## 5. PLR—Platelet to Lymphocyte Ratio

The predictive value of PLR stems from the important role platelets play in inflammation during cancer development. Platelets release vascular endothelial growth factor (VEGF) and platelet-derived growth factor (PDGF), which results in the induction of tumor growth, metastasis, and angiogenesis. Furthermore, platelets participate in tumor-induced thrombosis. This enables circulating tumor cells to evade T-lymphocyte-mediated immune surveillance and successfully extravasate to distant sites under the protection of platelets [26]. Lymphocytes, on the other hand, provide immunological protection, so an increased platelet count and a decreased lymphocyte count result in a high PLR, predicting a poorer patient prognosis. The study by Ikuta et al. turned out to be the only one in which the AUC value was good (0.713) with a sensitivity of 81% and a specificity of 58.7% of PLR as a predictor of mortality in patients with pancreatic cancer. Additionally, they found that the combined assessment of inflammatory markers, such as PLR, and the classic tumor marker CA19-9 may be an even better marker of early metastases after macroscopic resection in patients with PDAC. Elevated values of both parameters were associated with a more aggressive disease course and shorter survival, suggesting that PLR may serve as a complementary marker to CA19-9 in clinical risk stratification [60].

Similarly, analyses by Bao Dong et al. demonstrated good diagnostic value of the PLR index (0.703) with a sensitivity of 50% and a specificity of 80% for PLR as an indicator of overall survival in patients with pancreatic cancer and type 2 diabetes. The authors demonstrated that an elevated PLR (>126.42) predicts a significantly poorer response to treatment and poorer survival [61] (Table 3). These results suggest that the metabolic status of the patient may modulate the prognostic significance of PLR in pancreatic cancer. Iwai et al. demonstrated moderate prognostic value of PLR in predicting prognosis in patients with inoperable pancreatic cancer (AUC = 0.631). Patients with PLR values ≥146 had significantly shorter overall survival compared to those with PLR <146, especially when combined with an elevated CRP/Alb ratio and a reduced prognostic nutritional index (PNI). The authors noted that PLR remains indirectly dependent on other inflammatory and nutritional parameters and, therefore, should not be used as a standalone prognostic marker, and its usefulness requires further prospective studies [41]. Riauka et al. observed that elevated preoperative PLR values were associated with poorer overall survival, suggesting that inflammation and platelet activation may play a role in prognosis after tumor resection. These results indicate that PLR may be a useful, readily available complementary parameter in preoperative risk stratification [62]. Oh et al. demonstrated that a PLR score >150 was significantly associated with shorter overall survival in patients with pancreatic cancer. However, after adjusting for other clinicopathological factors, PLR did not always retain independent prognostic value [50]. All of the above observations are presented in Table 3.

Li W et al. demonstrated that a higher PLR score (>123) was associated with shorter overall survival in patients with pancreatic cancer. However, this indicator did not demonstrate as stable prognostic value as NLR. These results suggest that the prognostic significance of PLR depends on the stage of the disease and the treatment administered [26]. Shirai Y. et al. demonstrated that a high PLR score (>150) was significantly associated with an increased risk of disease recurrence and shorter overall survival in patients after pancreatic cancer resection. The authors emphasized the advantages of PLR as a readily available and cost-effective prognostic biomarker, routinely measured in complete blood counts. It has also been suggested that PLR may be helpful in identifying patients at higher risk of adverse disease outcomes who could potentially benefit from more intensive adjuvant treatment [63]. Zhou Y et al., in a meta-analysis of 17 cohort studies, demonstrated that a high PLR score (>150) is associated with poorer overall survival in patients with pancreatic cancer. Subgroup analyses, however, indicated that the prognostic significance of PLR was limited primarily to Asian populations and patients treated with combination therapy, confirming that the value of this score is dependent on the stage of disease and the treatment used. The authors further emphasized that PLR has a weaker and less stable prognostic value than NLR. However, further studies are needed to standardize this score [64]. Sakamoto et al. demonstrated that PLR scores >129.1 are significantly associated with a poorer prognosis and a more severe disease course. Importantly, the prognostic significance of PLR depends on clinical conditions and is primarily revealed in specific situations, such as advanced disease stage, combined treatment, or in combination with the classic tumor marker CA19-9 [65]. Studies by Giakoustidis A et al. demonstrated that PLR >120 is associated with shorter overall survival in patients with resectable pancreatic cancer. These results indicate that PLR may have prognostic significance, although its utility is limited compared to other inflammatory markers [66]. However, Pointer et al. did not demonstrate a significant association with overall or relapse-free survival in multivariate analyses, indicating limited utility of PLR as a prognostic marker in this specific patient population (add citation). Chen et al. did not confirm a clear, independent prognostic value of PLR for overall survival. These results suggest limited usefulness of PLR as a stand-alone prognostic marker in the analyzed cohort [52,67].

Elevated PLR may reflect enhanced platelet-mediated tumor growth, angiogenesis, metastatic potential, and immune evasion accompanied by reduced lymphocyte-driven antitumor activity. These mechanisms provide a biological rationale for the association between increased PLR and poorer prognosis observed in many clinical studies.

Pancreatic cancer remains one of the most aggressive solid tumors, with a very poor prognosis and limited therapeutic options. The course of the disease and the effectiveness of treatment are largely determined by complex interactions between cancer cells and the tumor microenvironment, in which inflammatory processes and immunosuppressive mechanisms play a key role. The tumor microenvironment of pancreatic cancer is characterized by high cellular heterogeneity, the dominance of stromal elements, and the presence of numerous immune cell populations with a phenotype conducive to tumor progression. Chronic inflammation promotes the activation of signaling pathways responsible for proliferation, angiogenesis, invasion, and immune evasion, which consequently leads to rapid disease progression and the development of treatment resistance. Available data indicate that the systemic inflammatory response is reflected in peripheral blood count parameters. Simple inflammatory markers, such as the neutrophil-to-lymphocyte ratio (NLR), lymphocyte-to-monocyte ratio (LMR), and platelet-to-lymphocyte ratio (PLR), may be useful tools for assessing prognosis in patients with pancreatic cancer. In most of the studies analyzed, elevated NLR and PLR values and decreased LMR correlated with a more aggressive disease course, shorter overall survival, and poorer response to anticancer treatment. Of the parameters discussed, NLR appears to be the most studied and stable prognostic marker, and its clinical value increases when combined with established biomarkers such as CA 19-9. The interpretation of the available evidence should be approached with caution because the included studies were characterized by substantial heterogeneity in patient populations, disease stage, treatment modalities, sample size, study design, clinical endpoints, and cut-off values used for inflammatory indices. These differences may limit direct comparisons across studies, affect reproducibility, and reduce the generalizability of the findings. In particular, the marked variability in the cut-off values reported for NLR, LMR, and PLR remains a major barrier to the standardization and routine clinical implementation of these biomarkers. Therefore, establishing validated and universally accepted thresholds should be a priority for future prospective research.

Another important limitation of the available evidence is that most included studies were retrospective observational analyses. Such study designs are inherently vulnerable to selection bias, confounding, and incomplete adjustment for factors that may affect inflammatory markers. Furthermore, inflammatory indices such as NLR, LMR, and PLR may be influenced by factors unrelated to cancer progression, including infections, autoimmune diseases, chronic inflammatory conditions, nutritional status, and concurrent treatments. Since these potential confounding factors were not consistently reported or adjusted for across the included studies, their impact on the observed prognostic associations cannot be excluded. Consequently, the reported associations between NLR, LMR, and PLR and clinical outcomes should be interpreted with caution until confirmed in large prospective studies incorporating standardized assessment and adjustment for these factors.

The methodological and clinical variability among the available studies also limits the feasibility of robust quantitative synthesis. Future systematic reviews and meta-analyses based on more standardized and homogeneous datasets may provide more precise estimates of the prognostic value of NLR, LMR, and PLR in pancreatic cancer.

From a clinical perspective, inflammation-based hematological indices may be particularly useful as adjunctive tools for prognostic stratification and treatment planning in patients with pancreatic cancer. Among the studied parameters, NLR currently demonstrates the strongest and most consistent prognostic evidence, especially when interpreted in conjunction with established biomarkers such as CA 19-9. Elevated NLR and PLR, as well as reduced LMR, may help identify patients with a more aggressive disease phenotype, poorer expected survival, or a potentially less favorable response to systemic therapy. However, these indices should not be considered substitutes for established diagnostic, laboratory, imaging, or molecular markers. Rather, due to their low cost, wide availability, and ease of assessment from routine complete blood count testing, they may serve as complementary biomarkers that enhance risk stratification and support clinical decision-making when integrated with conventional prognostic and diagnostic approaches. Future prospective studies are required to define their precise role within standardized diagnostic and therapeutic algorithms for pancreatic ductal adenocarcinoma.

In summary, inflammatory markers of peripheral blood counts represent promising, inexpensive, and readily available prognostic biomarkers in patients with pancreatic cancer. Their integration with clinical, laboratory, and molecular assessments may in the future contribute to better patient stratification, more accurate prognosis assessment, and personalized treatment. However, further prospective studies on large cohorts of patients are necessary to standardize the analyzed parameters and determine their actual place in diagnostic and therapeutic algorithms for PDAC.

## Figures and Tables

**Table 2:** LMR in Various Studies.

Authors	Study Group N	Control Group N	NLR Values		*p* Value	Cut-Off	AUC	Specificity (%)	Sensitivity (%)	Observations
			Study	Control						
Maloney Sara et al. 2023 [43]	Received neoadjuvant chemotherapy = 110	Surgery patients = 86	>3	<3	0.019	2.4	-	-	-	- A low preoperative LMR <2.4 was independently associated with poorer overall survival after curative resection of PDAC. - LMR showed prognostic but not predictive value, as it was not associated with response to adjuvant chemotherapy.
Stotz et al. 2015 [54]	N = 157	N = 86	>2.8	<2.8	0.004	2.8	0.603	58.82	60.34	- A low preoperative LMR <2.8 was associated with significantly poorer overall survival in patients undergoing resection for PDAC. - LMR remained an independent prognostic factor in multivariate analysis, reflecting an unfavorable immune-inflammatory balance.
Li et al. 2017 [53]	N = 969	N = 352	>3	<3	<0.001	3	-	-	-	- Patients with a lower preoperative LMR (3.0 experienced inferior overall survival following curative resection of pancreatic ductal adenocarcinoma. - After adjustment for clinicopathological factors, LMR retained independent prognostic relevance, while showing no association with adjuvant treatment response.
Lin et al. 2020 [55]	N = 778	N = 2560	>3	<3	<0.001	3	-	-	-	- Higher pretreatment LMR (>3) was significantly associated with improved overall survival in patients with pancreatic cancer (pooled HR <1). - Elevated LMR was also linked to better disease-free/recurrence-free survival, with consistent effects across different treatment modalities, ethnic groups, and LMR cut-off values.
Shimizu et al. 2019 [56]	N = 46	N = 37	<3.4	>3.4	<0.001	3.4	-	-	-	- A low baseline LMR (<3.4) was independently associated with poorer overall survival in patients with unresectable pancreatic cancer receiving chemotherapy. - LMR retained prognostic significance in multivariate analysis, indicating that immune–inflammatory status influences outcomes even in advanced, non-resectable disease.
Li et al. 2024 [57]	N = 911	N = 3108	>3	<3	<0.0001	3	-	-	-	- High pretreatment LMR was significantly associated with improved overall survival in resectable pancreatic cancer (pooled HR 0.55, 95% CI 0.44–0.69; *p* < 0.00001), with consistent findings across LMR thresholds (<3 or ≥3), tumor subtypes, and geographic regions. - LMR cutoff values had robust prognostic significance regardless of the threshold used (both <3 and ≥3), while no significant association was found with recurrence-free survival (HR 0.35, *p* = 0.12)
Pointer et al. 2020 [52]	N = 140	N = 137	>2.9	<2.9	NS	2.9	-	-	-	- LMR was not significantly associated with overall or recurrence-free survival after resection of early-stage pancreatic cancer.
Hu et al. 2018 [58]	N = 1192	N = 1365	>3	<3	<0.001	2.05–4.62	-	-	-	- Low LMR (<3) was associated with poorer overall survival and advanced disease features in pancreatic cancer, while showing greater prognostic stability than other inflammation-based indices.
Xue et al. 2017 [59]	Chinese Training Set N = 84	Chinese Training Set N = 69	>2.8	<2.8	<0.001	2.8	-	-	-	- Low preoperative LMR (<2.8) was independently associated with poorer overall survival after pancreatic cancer resection, confirming LMR as a reproducible and externally validated prognostic marker.
	Japanese Validation Set N = 106	Japanese Validation Set N = 146	>2.8	<2.8	0.005	2.8	-	-	-	

**Table 3:** PLR in Various Studies.

Authors	Study Group N	Control Group N	PLR Values		*p* Value	Cut-Off	AUC	Specificity (%)	Sensitivity (%)	Observations
			Study	Control						
Riauka et al. 2020 [62]	N = 9	N = 5	<150	>150	<0.00001	150	-	-	-	- PLR may serve as a predictive factor of better DFS in patients with resectable pancreatic cancer. - However, available evidence does not support PLR as a reliable prognostic factor for OS.
Oh et al. 2018 [50]	N = 4	N = 9	>150	<150	<0.001	150	0.490	-	-	- High PLR (>150) was associated with shorter overall survival in patients who had mixed treatment.
Iwai et al. 2020 [41]	N = 49	N = 69	<146	>146	0.002	146	0.631	-	-	- High PLR is prognostic factor for overall survival in patients with advanced pancreatic cancer, alongside CRP/Alb ratio and PNI.
Li et al. 2019 [26]	N = 77	N = 57	>123	<123	0.007	123	0.586	-	-	- An in vivo study has shown that anti-platelet drugs could reduce metastasis in PDAC.
Shirai et al. 2015 [63]	N = 73	N = 58	>150	<150	0.014	150	-	-	-	- High PLR (>150) was associated with increased recurrence risk and shorter overall survival after pancreatic cancer resection.
Zhou et al. 2018 [64]	N = 1882	N = 1300	>150	<150	<0.00001	150	-	-	-	- High PLR (>150) was associated with poorer overall survival in pancreatic cancer, with prognostic significance mainly observed in Asian populations and in patients receiving combined treatment.
Ikuta et al. 2019 [60]	N = 55	N = 58	>144	<144	0.001	144	0.713	58.7	81	- PLR was associated with survival in pancreatic cancer but showed limited and inconsistent independent prognostic value compared with other inflammatory indices. - A combination of PLR and CA19-9 is predictor of early recurrence after macroscopic resection for PDAC.
Sakamoto et al. 2019 [65]	N = 55	N = 49	>129.1	<129.2	0.049	129.1	0.560	-	-	- Elevated PLR is frequently associated with poorer overall survival in pancreatic cancer, although its prognostic value is generally weaker and less consistent than that of NLR. - The prognostic relevance of PLR is context-dependent, showing greatest utility in selected patient subgroups or when combined with CA19-9 as a complementary biomarker.
	Tumor size (>29.4 mm) N = 47	Tumor size (<29.4 mm) N = 54	170.9	131.1	0.002		-	-	-	
Alexandros Giakoustidis et al. 2018 [66]	N = 93	N = 34	>120	<120	0.038	120	-	-	-	- PLR >120 was associated with poorer overall survival in pancreatic cancer patients, although its prognostic value was weaker than that of NLR.
B. Dong and R.-R. Wu 2022 [61]	PC Patients with Type 2 diabetes N = 46	Healthy controls N = 50	134.13	108.32	<0.001	126.42	0.706	84	50	- Elevated PLR was associated with poorer overall survival in patients with pancreatic cancer and diabetes mellitus, indicating that metabolic status may modify the prognostic value of PLR.
Pointer et al. 2020 [52]	N = 138	N = 139	>144.4	<144.4	NS	192.6	-	-	-	- PLR was not significantly associated with overall or recurrence-free survival in patients with resected early-stage pancreatic cancer.
Chen Y et al. 2020 [67]	N = 39	N = 56	>169	<169	NS	169	-	-	-	- PLR did not demonstrate a consistent independent prognostic value for overall survival in pancreatic cancer.

## Data Availability

No new data were created or analyzed in this study. Data sharing is not applicable to this article.

## Abbreviations

CBC	complete blood count
NLR	neutrophil-to-lymphocyte ratio
LMR	lymphocyte-to-monocyte ratio
PLR	platelet-to-lymphocyte ratio
CAFs	cancer-associated fibroblasts
ECM	extracellular matrix
MMPs	metalloproteinases
VEGF	vascular endothelial growth factor
PDAC	pancreatic ductal adenocarcinoma
TAMs	tumor-associated macrophages
TANs	tumor-associated neutrophils
TAPs	tumor-associated platelets
MDSCs	myeloid-derived suppressor cells
DCs	dendritic cells
NK	natural killer
TLS	tertiary lymphoid structures
TCIPA	tumor cell-induced platelet aggregation
CLEC-2	C-type lectin receptor 2
TIMP3	tissue inhibitor of metalloproteinases 3
TLR	toll-like receptor
DAMPs	damage-associated molecular patterns
COX-2	cyclooxygenase-2
ROS	reactive oxygen species
RNS.	reactive nitrogen species
PDGF	platelet-derived growth factor
